# The complete mitochondrial genome of *Dysgonia stuposa* (Lepidoptera: Erebidae) and phylogenetic relationships within Noctuoidea

**DOI:** 10.7717/peerj.8780

**Published:** 2020-03-16

**Authors:** Yuxuan Sun, Yeshu Zhu, Chen Chen, Qunshan Zhu, Qianqian Zhu, Yanyue Zhou, Xiaojun Zhou, Peijun Zhu, Jun Li, Haijun Zhang

**Affiliations:** College of Life Sciences, Huaibei Normal University, Huaibei, Anhui, China

**Keywords:** Phylogenetic relationship, *D. stuposa*, Mitochondrial genome, Noctuoidea

## Abstract

To determine the *Dysgonia stuposa* mitochondrial genome (mitogenome) structure and to clarify its phylogenetic position, the entire mitogenome of *D. stuposa* was sequenced and annotated. The *D. stuposa* mitogenome is 15,721 bp in size and contains 37 genes (protein-coding genes, transfer RNA genes, ribosomal RNA genes) usually found in lepidopteran mitogenomes. The newly sequenced mitogenome contained some common features reported in other Erebidae species, e.g., an A+T biased nucleotide composition and a non-canonical start codon for *cox1* (CGA). Like other insect mitogenomes, the *D. stuposa* mitogenome had a conserved sequence ‘ATACTAA’ in an intergenic spacer between *trnS2* and *nad1*, and a motif ‘ATAGA’ followed by a 20 bp poly-T stretch in the A+T rich region. Phylogenetic analyses supported *D. stuposa* as part of the Erebidae family and reconfirmed the monophyly of the subfamilies Arctiinae, Catocalinae and Lymantriinae within Erebidae.

## Introduction

*Dysgonia stuposa* (Lepidoptera: Erebidae) is an important pest species, and it has a wide distribution throughout the southern and eastern parts of Asia. Its larvae mainly consume the leaves of *Punica granatum* (pomegranate) resulting in considerable economic losses. In the northern areas of China, *D. stuposa* pupates during the winter to avoid the harsh environment (Piao, Fan & Zheng, 2012). The identification and prevention of *D. stuposa* at the pupal stage based on morphological characteristics is quite difficult for taxonomists and population ecologists. Despite the economic importance, our understanding of *D. stuposa* biology or phylogenetic status at the molecular level is still in its infancy. New molecular techniques such as DNA barcoding and PCR-RFLP are considered more reliable than morphology for studying taxonomy of animals (Arimoto & Iwaizum, 2014; Raupach et al., 2010). The application of molecular techniques to study the sequence of *D. stuposa* mitogenome will help in its precise identification and classification while contributing to future genetic ecology and evolutionary analyses.

The insect mitogenome is typically a 14–19 kb sized, circular, double-stranded DNA molecule (Boore, 1999). Compared to the nuclear genome, mitogenome is small in size and comparatively easy to sequence. Mitogenome usually contains numerous typical characteristics, such as stable gene composition, and conserved gene arrangements, which are widely used in molecular identification, population genetics, systematics and biogeographic studies (Wolstenholme, 1992; Wilson et al., 2010). Given the vast diversity of insects, mitogenome analyses are beneficial for species identification and broadly employed in the study of genomic evolution and phylogenetic relationships (Lu et al., 2013; Cameron, 2014).

Noctuoidea is one of the largest superfamilies of Lepidoptera, with over 42,400 described species (Nieukerken et al., 2011). Unlike other superfamilies, a metathoracic tympanal organ is a characteristic feature of Noctuoidea species (Miller, 1991). However, morphological based phylogenetics has failed to resolve classification conflicts at the family and sub-family level. Furthermore, the initial molecular studies were also unable to provide sufficient information as most of them rely on one or two genes with only 29–49 species (Mitchell et al., 1997; Fang et al., 2000). Mitchell, Mitter & Regier (2006) conducted systemic analyses based on two nuclear genes (*elongation factor-1α* (*EF-1α*) and *dopa decarboxylase* (*DDC*)) and increased taxon sampling (146 species), that supported the monophyly of sub-families and proposed a LAQ clade (Lymantriidae and Arctiidae became subordinate subfamilies within quadrifid noctuids). Zahiri et al. (2011) reconstructed the molecular phylogenetics of Noctuoidea using one mitochondrial (*cox1*) and seven nuclear genes *(EF-1α*, *wingless*, *RpS5*, *IDH*, *CMDH*, *GAPDH* and *CAD*) from 152 species with the Maximum Likelihood (ML) method. They proposed a new perspective, splitting up the traditional group of quadrifid noctuids, and re-establishing Erebidae and Nolidae as families (Zahiri et al., 2011). However, this study failed to clarify phylogenetic relationships between Erebidae subfamilies (Zahiri et al., 2012). Additionally, morphological studies were not entirely consistent with the molecular studies in challenging some traditional synapomorphies, such as the “quadrifid” forewing venation and the presence of a transverse sclerite in the pleural region of segment A1 (Minet, Barbut & Lalanne-Cassou, 2012).

Complete mitogenomes and the mitochondrial genes are increasingly applied to understand phylogenetic relationships. For example, Wang et al. (2015) proposed two new tribes and established relationships between them within Lymantriinae by using two mitochondrial genes (*cox1* and *rrnL*) along with six nuclear genes, using ML and Bayesian Inference (BI). The nucleotide and amino acid sequences of mitochondrial PCGs are also broadly used to determine the taxonomic status of species and to analyze phylogenetic relationships within Erebidae (Yang & Kong, 2016; Liu et al., 2017). Furthermore, as the mitogenome differs from the nuclear genome, it has been increasingly used to investigate poorly supported phylogenetic questions such as the position of Nymphalidae within Papilionoidea (Yang et al., 2009). Since many species of the genus *Dysgonia* have been moved to other genera, including Erebidae and Noctuidae based on the classification of Holloway & Miller (2003), the taxonomic status of many species remained uncertain. In our study, we sequenced the complete mitogenome of *D. stuposa* and reconstructed phylogenetic relationships to assess its phylogenetic position within Noctuoidea. The newly sequenced mitogenome supported new phylogenetic relationships within Erebidae and will provide a foundation for further studies into Noctuidae and Erebidae mitogenomics, biogeography, and phylogenetics.

## Material and Methods

### Specimen collection and Genomic DNA extraction

The *D. stuposa* moths were collected from Xiangshan mountains (N33°59′, E116°47′), Huaibei, Anhui, China. Based on morphological characteristics, the collected specimens were identified as *D. stuposa* using the record in *Fauna Sinica* (Chen, 2003). The genomic DNA (contains nuclear genome and mitogenome) of *D. stuposa* was isolated using the Animal Genomic DNA Isolation Kit according to the manufacturer’s instructions (Sangon, Shanghai, China).

### PCR amplification and fragment sequencing

To amplify the *D. stuposa* mitogenome, the universal (F1-R13) and specific primers (S1F-S3R) were used to perform PCR amplification (Table 1) (Sun et al., 2016). All PCR amplifications were executed using high fidelity DNA Polymerase (PrimeSTAR^®^ GXL, Takara, Dalian, China). PCRs was performed according to Sun et al. (2016) with extension times depending on the putative length of target fragment. PCR product size was determined by agarose gel with TAE buffer, then sequenced at General Biosystems (General, Chuzhou, China) in both forward and reverse directions using ABI 3500 Genetic Analyzer by the Sanger sequencing method. For long fragments, internal sequencing primers were designed based on known fragment sequence. For the A+T rich region, the fragment was sequenced from two directions and repeated three times.

**Table 1:** Details of the primers used to amplify the mitochondrial DNA of *D. stuposa*.

Primer name	Nucleotide sequence (5′–3′)
F1	TAAAAATAAGCTAAATTTAAGCTT
R1	TATTAAAATTGCAAATTTTAAGGA
F2	AAACTAATAATCTTCAAAATTAT
R2	AAAATAATTTGTTCTATTAAAG
F3	ATTCTATATTTCTTGAAATATTAT
R3	CATAAATTATAAATCTTAATCATA
F4	TGAAAATGATAAGTAATTTATTT
R4	AATATTAATGGAATTTAACCACTA
F5	TAAGCTGCTAACTTAATTTTTAGT
R5	CCTGTTTCAGCTTTAGTTCATTC
F6	CCTAATTGTCTTAAAGTAGATAA
R6	TGCTTATTCTTCTGTAGCTCATAT
F7	TAATGTATAATCTTCGTCTATGTAA
R7	ATCAATAATCTCCAAAATTATTAT
F8	ACTTTAAAAACTTCAAAGAAAAA
R8	TCATAATAAATTCCTCGTCCAATAT
F9	GTAAATTATGGTTGATTAATTCG
R9	TGATCTTCAAATTCTAATTATGC
F10	CCGAAACTAACTCTCTCTCACCT
R10	CTTACATGATCTGAGTTCAAACCG
F11	CGTTCTAATAAAGTTAAATAAGCA
R11	AATATGTACATATTGCCCGTCGCT
F12	TCTAGAAACACTTTCCAGTACCTC
R12	AATTTTAAATTATTAGGTGAAATT
F13	TAATAGGGTATCTAATCCTAGTT
R13	ACTTAATTTATCCTATCAGAATAA
S1F	ACTTTAAAAACTTCAAAGAAAAA
S1R	ACTTAATTTATCCTATCAGAATAA
S2F	CGCAACTGCTGGCACAAA
S2R	GAAGAGAAGTTTATAGTGGATGAGGTT
S3F	TAAGCTGCTAACTTAATTTTTAGT
S3R	GTAATAAATTCCTCGTCCAATAT

**Table 2:** Details of the lepidopteran mitogenomes used in this study.

Family	Subfamily	Species	Size (bp)	GenBank No.
Erebidae	Arctiinae	*Spilarctia subcarnea*	15,441	KT258909
		*Lemyra melli*	15,418	KP307017
		*Hyphantria cunea*	15,481	GU592049
		*Nyctemera arctata albofasciata*	15,432	KM244681
		*Callimorpha dominula*	15,496	KP973953
		*Aglaomorpha histrio*	15,472	KY800518
		*Amata formosae*	15,463	KC513737
		*Cyana sp. MT-2014*	15,494	KM244679
		*Paraona staudingeri*	15,427	KY827330
		*Vamuna virilis*	15,417	KJ364659
	Catocalinae	*Grammodes geometrica*	15,728	KY888135
		*Catocala sp. XY-2014*	15,671	KJ432280
		*Dysgonia stuposa*	15,721	This study
	Herminiinae	*Hydrillodes lentalis*	15,570	MH013484
	Aganainae	*Asota plana lacteata*	15,416	KJ173908
	Hypeninae	*Paragabara curvicornuta*	15,532	KT362742
	Lymantriinae	*Gynaephora minora*	15,801	KY688086
		*Gynaephora aureata*	15,773	KJ507132
		*Lachana alpherakii*	15,755	KJ957168
		*Gynaephora qumalaiensis*	15,753	KJ507134
		*Euproctis similis*	15,437	KT258910
		*Somena scintillans*	15,410	MH051839
Noctuidae	Noctuinae	*Agrotis ipsilon*	15,377	KF163965
		*Agrotis segetum*	15,378	KC894725
	Hadeninae	*Mythimna separata*	15,329	KM099034
		*Protegira songi*	15,410	KY379907
	Amphipyrinae	*Sesamia inferens*	15,413	JN039362
		*Spodoptera exigua*	15,365	JX316220
		*Spodoptera litura*	15,383	KF701043
		*Spodoptera frugiperda*	15,365	KM362176
	Heliothinae	*Helicoverpa armigera*	15,347	GU188273
		*Helicoverpa zea*	15,343	KJ930516
		*Helicoverpa assulta*	15,400	KT626655
		*Heliothis subflexa*	15,323	KT598688
	Plusiinae	*Ctenoplusia agnata*	15,261	KC414791
		*Ctenoplusia limbirena*	15,306	KM244665
Nolide	Chloephorinae	*Gabala argentata*	15,337	KJ410747
	Risobinae	*Risoba prominens*	15,343	KJ396197
Notodontidae	Thaumetopoeinae	*Ochrogaster lunifer*	15,593	AM946601
	Phalerinae	*Phalera flavescens*	15,659	JF440342
outgroup		*Bombyx mori*	15,664	AY048187
outgroup		*Antheraea pernyi*	15,566	AY242996

### Sequence assembly and annotation

The complete mitogenome was assembled using the DNAMAN (https://www.lynnon.com/index.html). Sequence annotation (supplied in supplemental files) was performed by MITOS2 Web Server (http://mitos2.bioinf.uni-leipzig.de/index.py) and confirmed by BLAST to homologous sequences in NCBI (https://blast.ncbi.nlm.nih.gov/Blast.cgi). To determine PCG initiation and termination codons, sequences were aligned with other published Noctuoidea sequences using ClustalX 2.0 (Larkin et al., 2007). AT skew and GC skew values were calculated using the methods given by Perna & Kocher (1995). MEGA 5.0 software was used to analyze relative synonymous codon usage (RSCU) (Tamura et al., 2011). tRNA genes were determined by tRNAscan Search Server (http://lowelab.ucsc.edu/tRNAscan-SE/) and secondary structures inferred from folding into their canonical clover-leaf structures (Lowe & Eddy, 1997). rRNA genes were determined by MITOS2 Web Server and confirmed by BLAST with the homologous sequences in NCBI. Tandem Repeats Finder (http://tandem.bu.edu/trf/trf.html) was used to analyze non-coding regions for tandem repeats (Benson, 1999).

### Phylogenetic analysis

To infer the phylogenetic relationships among Noctuoidea at superfamily level, concatenated nucleotide sequence alignments for PCGs from 42 species (Table 2) was performed. All of the sequences were downloaded from GenBank. The Saturnidae species *Bombyx mori* (AY048187) and *Antheraea pernyi* (AY242996) (Liu et al., 2008) were used as outgroups. Sequences were aligned using ClustalX 2.0 software (Larkin et al., 2007). ML and BI were used to reconstruct phylogenetic relationships. For the ML analysis, nucleotide sequences were partitioned and performed in IQ-TREE (http://iqtree.cibiv.univie.ac.at/) with the best-fit model GTR+F+I+G4 (Trifinopoulos et al., 2016), and the clade support was investigated with 1000 bootstrap replicates. For the BI analysis, the GTR model and Invgamma rate variation across sites were presented and performed with MrBayes 3.2.6 (Ronquist et al., 2012). One cold chain and three heated chains were run with the dataset for 10 million generations with the tree being sampled every 1,000 generations. After discarding the first 25% samples as burn-in, posterior probabilities were calculated. The phylogenetic trees were visualized in FigTree software (http://tree.bio.ed.ac.uk/software/figtree/).

## Results and Discussion

### Genome organization and composition

**Figure 1:**
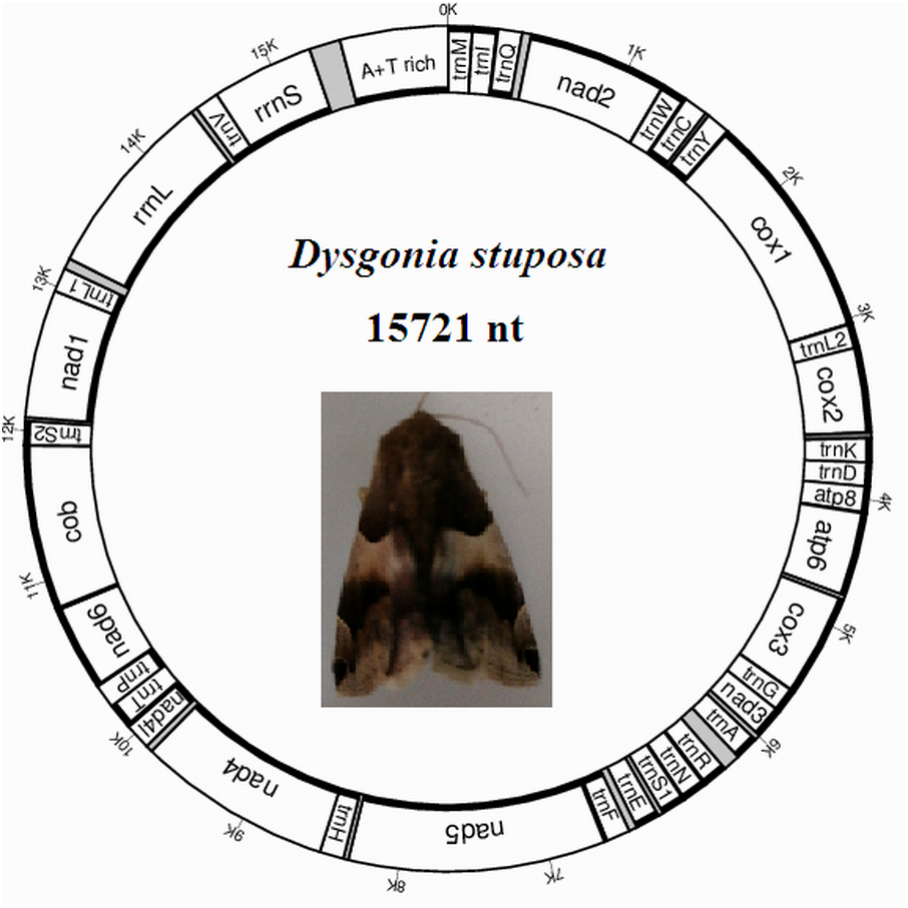
Map of the mitogenome of *D. stuposa*. tRNA genes are labeled according to the IUPAC-IUB one-letter amino acids; *cox1*, *cox2* and *cox3* refer to the cytochrome c oxidase subunits; *cob* refers to cytochrome b; *nad1-nad6* refer to NADH dehydrogenase components. The moth was photographed by the corresponding author Jun Li.

The *D. stuposa* mitogenome is a circular DNA molecule, which is 15,721 bp in length (accession number: MK262707) (Fig. 1). The size of the newly sequenced mitogenome is comparable to other Noctuoidea species, which range from 15,377 bp (*Agrotis ipsilon*) to 15,801 bp (*Gynaephora minora*) (Table 3). The variation in size is generally due to differences in the length of their non-coding regions (intergenic spacers and A+T rich region) (Lv, Li & Kong, 2018). Annotation found the typical 37 genes and a non-coding A+T rich region like most of the sequenced insect mitogenomes (Table 4). An A and T biased nucleotide composition is a characteristic feature of insect mitogenomes (Boore, 1999), and *D. stuposa* is no exception. Nucleotide composition of *D. stuposa* was highly biased towards using A and T (*A* = 39.98%, *T* = 40.38%, *G* = 7.5%, *C* = 12.14%) (Table 3); 80.36% total A+T content is comparable to previously sequenced lepidopterans (ranges from 77.84% in *Ochrogaster lunifer* to 81.49% in *Gynaephora minora*).

**Table 3:** Composition and skew in mitogenomes of Noctuoidea species.

Species	Size (bp)	A%	G%	T%	C%	A+T %	AT skew	GC skew
Whole genome								
*D. stuposa*	15,721	39.98	7.5	40.38	12.14	80.36	−0.005	−0.236
*A. plana lacteata*	15,416	40.08	7.49	40.26	12.16	80.34	−0.002	−0.238
*V.a virilis*	15,417	40.18	7.56	40.22	12.05	80.4	0.000	−0.229
*G. minora*	15,801	40.97	6.77	40.52	11.75	81.49	0.006	−0.269
*R. prominens*	15,343	40.25	7.8	40.82	11.13	81.07	−0.007	−0.176
*O. lunifer*	15,593	40.09	7.56	37.75	14.6	77.84	0.030	−0.318
*A. ipsilon*	15,377	40.38	7.71	40.87	11.04	81.25	−0.006	−0.178
PCGs								
*D. stuposa*	11,269	33.80	10.91	44.64	10.65	78.45	−0.138	0.012
*A. plana lacteata*	11,211	33.87	10.92	44.76	10.45	78.63	−0.138	0.022
*V. virilis*	11,203	33.14	11.16	45.43	10.27	78.57	−0.156	0.042
*G. minora*	11,237	34.72	10.11	44.98	10.2	79.7	−0.129	−0.004
*R. prominens*	11,216	33.64	10.57	46	9.8	79.64	−0.155	0.038
*O. lunifer*	11,266	32.47	12.08	43.26	12.19	75.73	−0.142	−0.005
*A. ipsilon*	11,211	34.24	10.64	45.56	9.55	79.8	−0.142	0.054
A+T rich								
*D. stuposa*	406	43.6	2.46	48.77	5.17	92.37	−0.056	−0.355
*A. plana lacteata*	328	46.04	1.22	48.48	4.27	94.52	−0.026	−0.556
*V. virilis*	362	44.48	1.1	50.55	3.87	95.03	−0.064	−0.557
*G. minora*	449	43.21	2.67	49.44	4.68	92.65	−0.067	−0.273
*R. prominens*	342	44.15	2.34	49.42	4.09	93.57	−0.056	−0.272
*O. lunifer*	319	44.51	1.57	48.9	5.02	93.41	−0.047	−0.524
*A. ipsilon*	332	46.08	1.51	48.8	3.61	94.88	−0.029	−0.410

**Table 4:** List of annotated mitochondrial genes of *D. stuposa*.

Gene name	Start	Stop	Strand	Length	Anti- codon	Start codon	End codon	Intergenic nucleotides
*trnM*	1	68	J	68	CAT	/	/	2
*trnI*	71	138	J	68	GAT	/	/	8
*trnQ*	147	215	N	69	TTG	/	/	55
*nad2*	271	1,284	J	1,014	/	ATT	TAA	−2
*trnW*	1,283	1,350	J	68	TCA	/	/	−8
*trnC*	1,343	1,409	N	67	GCA	/	/	22
*trnY*	1,432	1,496	N	65	GTA	/	/	9
*cox1*	1,506	3,041	J	1,536	/	CGA	TAA	−5
*trnL2*	3,037	3,103	J	67	TAA	/	/	0
*cox2*	3,104	3,820	J	717	/	ATA	TAA	−35
*trnK*	3,786	3,856	J	71	CTT	/	/	0
*trnD*	3,857	3,923	J	67	GTC	/	/	0
*atp8*	3,924	4,085	J	162	/	ATC	TAA	−7
*atp6*	4,079	4,756	J	678	/	ATG	TAA	27
*cox3*	4,784	5,572	J	789	/	ATG	TAA	2
*trnG*	5,575	5,640	J	66	TCC	/	/	0
*nad3*	5,641	5,994	J	354	/	ATT	TAA	34
*trnA*	6,029	6,085	J	57	TGC	/	/	105
*trnR*	6,191	6,256	J	66	TCG	/	/	10
*trnN*	6,267	6,332	J	66	GTT	/	/	8
*trnS1*	6,341	6,406	J	66	GCT	/	/	32
*trnE*	6,439	6,506	J	68	TTC	/	/	50
*trnF*	6,557	6,624	N	68	GAA	/	/	−17
*nad5*	6,608	8,368	N	1,761	/	ATT	TAA	−3
*trnH*	8,366	8,433	N	68	GTG	/	/	0
*nad4*	8,434	9,772	N	1,338	/	ATG	TA	42
*nad4l*	9,815	10,102	N	288	/	ATG	TAA	14
*trnT*	10,117	10,181	J	65	TGT	/	/	0
*trnP*	10,182	10,246	N	65	TGG	/	/	7
*nad6*	10,254	10,784	J	531	/	ATT	TAA	14
*cob*	10,799	11,959	J	1,161	/	ATG	TAA	−2
*trnS2*	11,958	12,025	J	68	TGA	/	/	22
*nad1*	12,048	12,986	N	939	/	ATG	TAA	1
*trnL1*	12,988	13,055	N	68	TAG	/	/	65
*rrnL*	13,121	14,428	N	1,308	/	/	/	37
*trnV*	14,466	14,533	N	68	TAC	/	/	0
*rrnS*	14,534	15,315	N	782	/	/	/	0
AT-rich region	15,316	15,721	/	406	/	/	/	/

AT ((A-T)/(A+T)) and GC skew ((G-C)/(G+C)) were calculated for the J strand (majority) (Perna & Kocher, 1995); with negative AT skew (−0.005) and GC skew (−0.236), indicating the presence of more Ts than As, and Cs than Gs, respectively (Table 3). Negative AT skew has been reported in several other insect species such as *Asota plana lacteata* (−0.002), *Risoba prominens* (−0.007) and *Agrotis ipsilon* (−0.006).

### Protein-coding genes and codon usage

PCGs identified from the *D. stuposa* mitogenome had a total length of 11,269 bp, accounting for 71.7% of the mitogenome. In insects, most PCGs are on the J strand (majority), while some of them reside on the N strand (minority) (Simon et al., 1994). In *D. stuposa*, nine of the thirteen PCGs (*nad2*, *cox1*, *cox2*, *atp8*, *atp6*, *cox3*, *nad3*, *nad6* and *cob*) are encoded on the J-strand, while the remaining PCGs (*nad5*, *nad4*, *nad4L* and *nad1*) are on the N-strand. An ATN codon initiated all PCGs except *cox1,* which uses a CGA codon, as in most Lepidoptera (Table 4). The utilize of non-canonical initiation codons for *cox1* is a common feature across insects (Liu et al., 2016; Dai et al., 2016).

**Figure 2:**
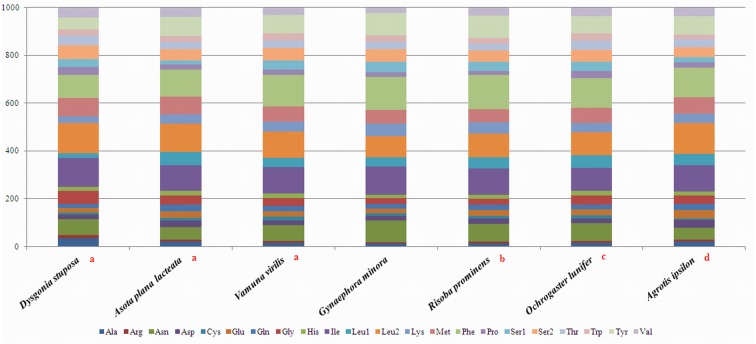
Comparison of codon usage within the mitochondrial genome of members of the Noctuoidea. Lowercase letters (a, b, c and d) above species names represent the family to which the species belongs (a: Erebidae, b: Nolide, c: Notodontidae, d: Noctuidae).

**Figure 3:**
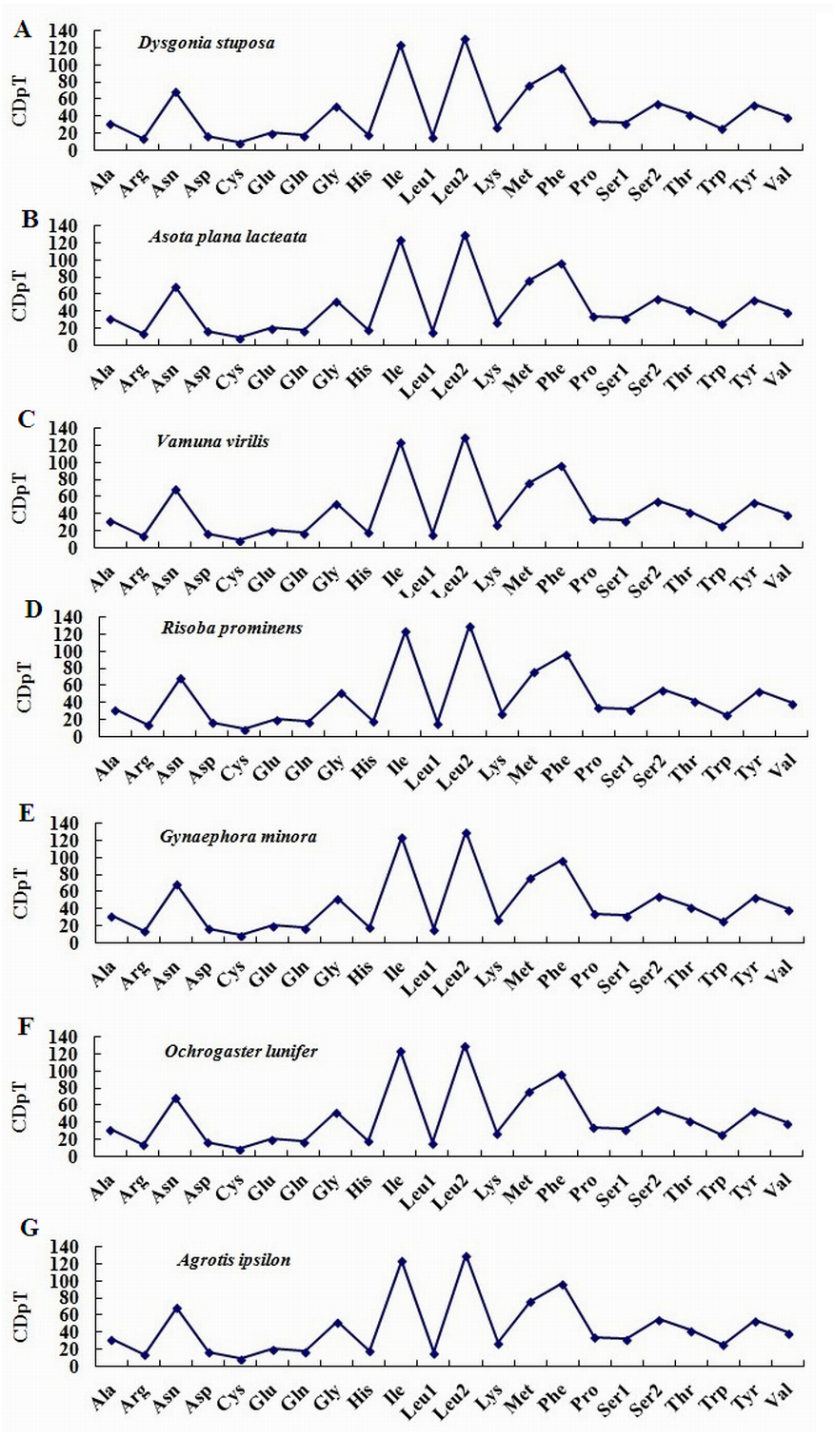
Codon distribution in members of the Noctuoidea. (A) CDspT of *Dysgonia stuposa*. (B) CDspT of *Asota plana lacteata*. (C) CDspT of *Vamuna virilis*. (D) CDspT of *Risoba prominens*. (E) CDspT of *Gynaephora minora*. (F) CDspT of *Ochrogaster lunifer*. (G) CDspT of *Agrotis ipsilon*. CDspT, codons per thousand codons.

To estimate codon usage among Noctuoidea species and to assess similarities and variations in codon usage and distribution, PCGs nucleotide sequences of seven Noctuoidea (belonging to four families: Erebidae, Noctuidae, Nolidae and Notodontidae) were compared (Fig. 2). In *D. stuposa* phenylalanine (Phe), asparagine (Asn), leucine (Leu), methionine (Met), tyrosine (Tyr) and isoleucine (Ile) were the most commonly used amino acids, while cysteine (Cys) was the most rarely utilized amino acid. Codon usage is similar across Noctuoidea. Furthermore, we used the codons per thousand (CDspT) metric to illustrate the codons distribution in different species (Dai et al., 2015) (Fig. 3). CDspT results exhibited similar trends across the Noctuoidea superfamily, with the maximum CDspT value observed for Asn and Ile.

**Figure 4:**
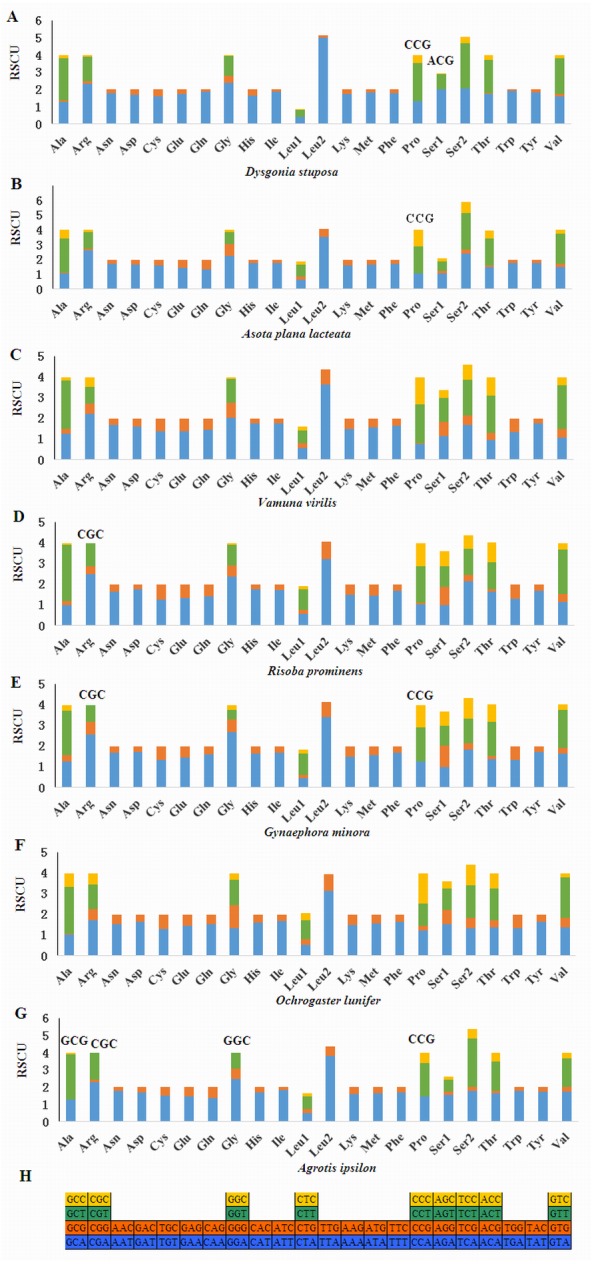
Relative Synonymous Codon Usage (RSCU) of the mitochondrial genome of four families in the Noctuoidea. (A) RSCU of *Dysgonia stuposa*. (B) RSCU of *Asota plana lacteata*. (C) RSCU of *Vamuna virilis*. (D) RSCU of *Risoba prominens*. (E) RSCU of *Gynaephora minora*. (F) RSCU of *Ochrogaster lunifer*. (G) RSCU of *Agrotis ipsilon*. (H) Codon families of synonymous codon. Codons indicated above the bar are not present in the mitogenome.

Relative Synonymous Codon Usage (RSCU) for Noctuoidea species is presented in Fig. 4. Codons usage within a given amino acid varied between species. All codons were found in *D. stuposa*, except ACG and CCG. Some noctuid species lack GC rich synonymous codons, with G or C at the third codon position, such as GCG, CGC, GGC and CCG (e.g., these are not present in *A. ipsilon*) (Wu, Cui & Wei, 2015). The rarity or complete absence of GC-rich codons occur in various insect species (Sun et al., 2017; Li et al., 2018).

**Figure 5:**
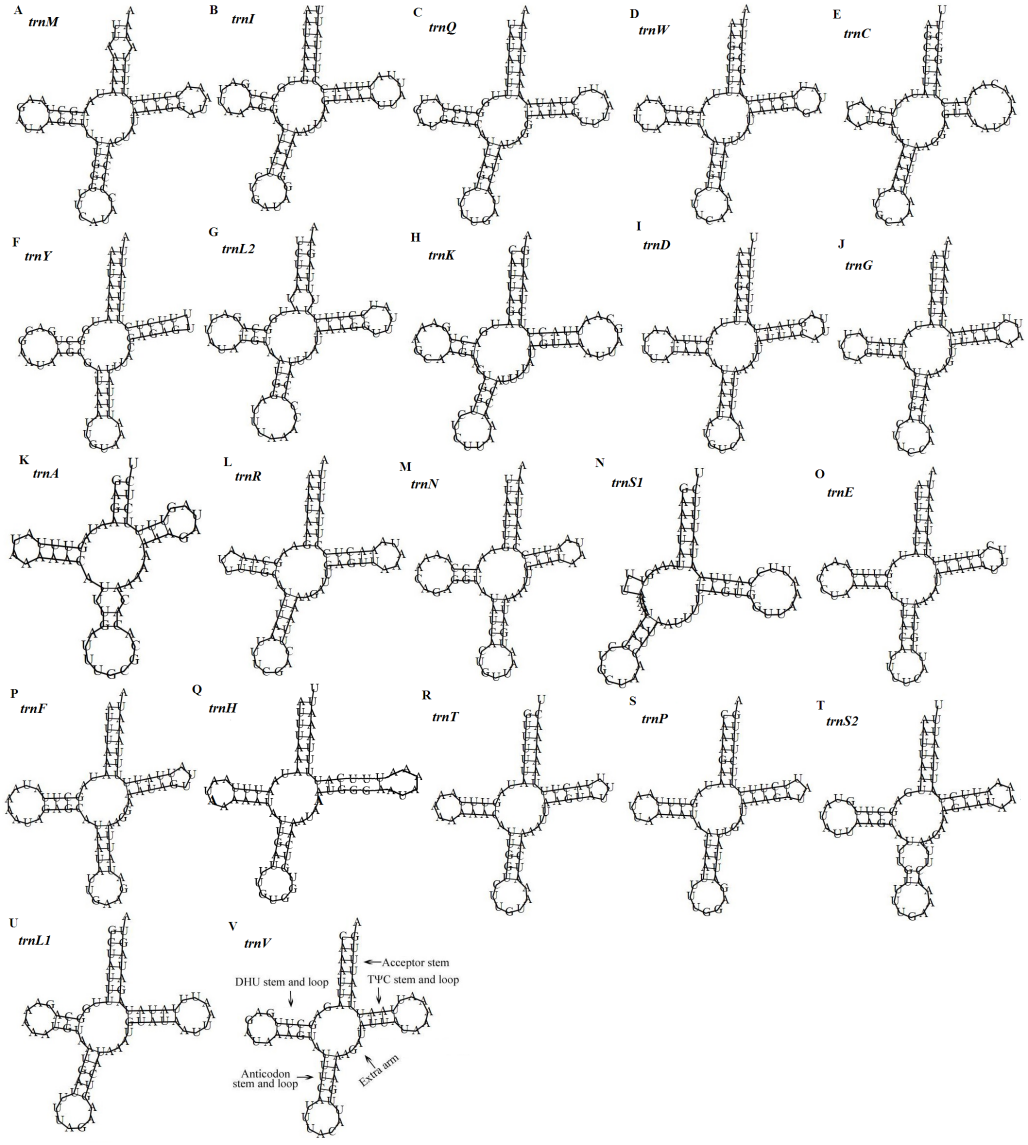
Predicted secondary structures of the 22 tRNA genes of the *D. stuposa* mitogenome. (A–V) Twenty-two tRNA secondary structures.

### Ribosomal RNA and transfer RNA genes

The *D. stuposa* mitogenome contains the large (*rrnL*) and small ribosomal genes (*rrnS*), encoded by the N strand with a length of 1,308 bp and 782 bp, respectively (Fig. 1, Table 4). In *D. stuposa*, *rrnL* was located between *trnL1* and *trnV*, while *rrnS* was resided between *trnV* and the AT-rich region, as reported in previously sequenced mitogenomes (Yang et al., 2009).

There are 22 tRNA genes in the *D. stuposa* mitogenome, ranging in size from 57 bp (*trnA*) to 71 bp (*trnK*) (Table 4). Almost all tRNAs had the canonical clover-leaf secondary structure, except *trnS1* that lacks the dihydrouridine (DHU) arm (Fig. 5), a common feature of *trnS1* across mitogenomes of insects (Lavrov, Brown & Boore, 2000; Zhang et al., 2013). Stem pair mismatches in the secondary structure of tRNAs were observed such as an A-A mismatch (*trnM*), U-G mismatches (*trn I*, *trnQ*, *trnW*, *trn Y*, *trnL2*, *trnG, trnF*, *trnH*, *trn T*, *trnP*, *trnV*), U-U mismatches (*trn Y*, *trnL2*, *trnS2*) and a U-C mismatch (*trnA*). These mismatches may be corrected by an RNA-editing process which was proposed by Lavrov, Brown & Boore (2000), but has not been investigated fully in Lepidoptera.

### Overlapping, intergenic spacer and A+T rich regions

**Figure 6:**
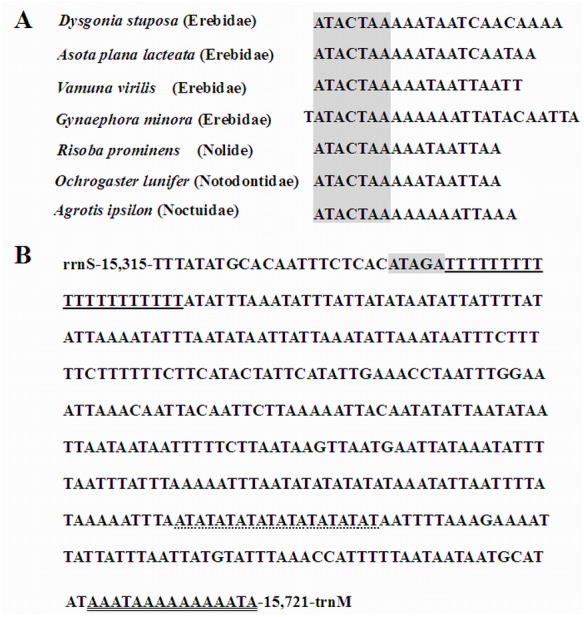
Features in the intergenic spacer and the A+T rich region. (A) Alignment of the intergenic spacer region between *trnS2* and *nad1* of several Noctuoidea insects. (B) Features present in the A+T-rich region of *D. stuposa.* The ‘ATAGA’ motif is shaded. The poly-T stretch is underlined and the poly-A stretch is double underlined. The single microsatellite ‘AT’ repeat sequence is indicated by dotted underlining.

Overlapping genes has been proposed to extend the genetic information possibly within the limited size of the genome, and are commonly observed in metazoan mitogenomes (Wolstenholme, 1992). We identified nine overlapping regions, a total length of 144 bp (Table 4). A seven bp overlapping region present at the boundary of *atp6* and *atp8* has also been reported in many other insects. The *D. stuposa* mitogenome also had 21 intergenic spacer regions, ranging in size from 1 to 105 bp. The 105 bp spacer located between *trnA* and *trnR* and had high A and T content (*A* = 47.62% and *T* = 49.52%) and a similar spacer has been described in *Andraca theae* (77 bp spacer with *A* = 46.75% and *T* = 44.16%). We also observed a 22 bp spacer that contained an ‘ATACTAA’ motif located between *nad1* and *trnS2* (Fig. 6A). This region commonly exists in most insect mitogenomes even though the region varies in size between lepidopteran species (Cameron & Whiting, 2008).

**Figure 7:**
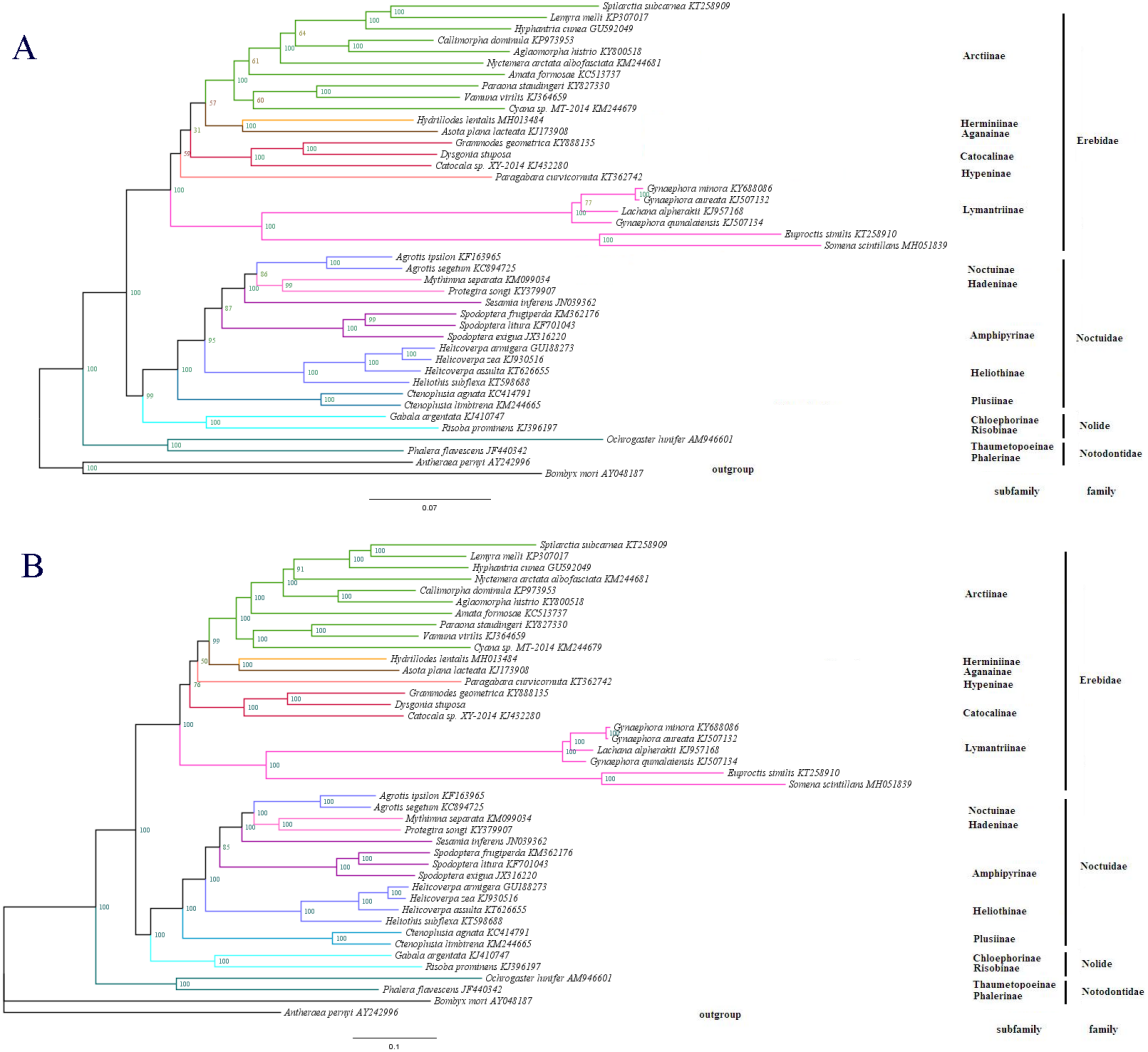
The phylogenetic relationships within Noctuoidea. (A) Tree showing the phylogenetic relationships among 43 species, constructed using Maximum Likelihood with 1000 bootstrap replicates. (B) Tree constructed using Bayesian Inference (BI) MCMC consensus tree, with posterior probabilities shown at nodes. *Bombyx mori* (AY048187) and *Antheraea pernyi* (AY242996) were used as outgroups.

Metazoan mitogenomes usually have a single large non-coding region, named as the A+T rich region (Clayton, 1991). It contains initiation signals for DNA transcription and replication (Fernández-Silva, Enriquez & Montoya, 2003). The A+T rich region of *D. stuposa* mitogenome is located between *rrnS* and *trnM* and is 406 bp in size (Table 4), with the negative GC skew (−0.355) and highest A+T content (92.37%) of the genome (Table 3). The A+T rich region usually contains multiple tandem repeat elements (Zhang & Hewitt, 1997); however, *D. stuposa* did not have macro-repeats but does include short repeating sequences. It has the ‘ATAGA’ motif along with a 20 bp poly-T repeat, a microsatellite-like (AT)_10_ repeat and a poly-A repeat sequence upstream of *trnM* (Fig. 6B). The poly-T stretch varies between different species (Dai et al., 2015), but the ‘ATAGA’ motif is conserved in insects (Zhang & Hewitt, 1997).

### Phylogenetic relationships

To determine the phylogenetic position of *D. stuposa*, we reconstructed phylogenetic relationships with Noctuoidea species. In phylogenetic analyses, mitogenome PCGs have a lower sensitivity to analytical bias compared to other genes such as the tRNA or rRNA genes (Yang et al., 2015). Here, we applied the nucleotide sequence of the 13 PCGs for phylogenetic analyses using BI and ML methods. Results showed that *D. stuposa* is closely related to *Grammodes geometrica*, a clade that was well supported by both the methods (Figs. 7A and 7B). *D. stuposa* belongs to the family Erebidae and subfamily Catocalinae, consistent with the reported classification of Erebidae (Zahiri et al., 2011). Erebidae is a large noctuid family (Yang et al., 2015); however, its monophyly remained unconfirmed, especially for Catocalinae (Zahiri et al., 2012). In the present study, the Catocalinae was found monophyletic, but nodal support values were not significant, i.e., 0.76 posterior probability (BI) and 31% bootstrap values (ML). There is still some controversy about relationships of Catocalinae under Erebidae. Zahiri et al. (2011) demoted Catocalinae to a tribe Catocalini within the subfamily Erebinae, and upgraded Anobini (formerly as a tribe within Catocalinae by Holloway (2005) to subfamily Anobinae. Several species of the *Dysgonia* genus have been reclassified into Noctuidae (Holloway & Miller, 2003), results in further complications for phylogenetic analysis. Within Erebidae, our study supported the monophyly of subfamilies and suggested that Catocalinae is a subfamily, most closely related to Hypeninae (BI) or Aganainae (ML) (Figs. 7A and 7B). Furthermore, Noctuoidea contained four families: Notodontidae, Erebidae, Nolidae and Noctuidae, for which their phylogenetic relationship was Notodontidae + (Erebidae + (Nolidae + Noctuidae)) with strong nodal support in both ML and BI trees. Since there is limited data of complete mitogenome sequences from Oenosandridae and Euteliidae in the public repository NCBI, our results are consistent with the previous family-level phylogenetic hypothesis proposed by Zahiri et al. (2011).

##  Supplemental Information

10.7717/peerj.8780/supp-1 Supplemental Information 1 The annotation of *D. stuposa* mitogenome

10.7717/peerj.8780/supp-2 Supplemental Information 2 The complete mitochondrial genome of *Dysgonia stuposa*

## Additional Information and Declarations

### Data Availability

The following information was supplied regarding data availability: The complete mitochondrial genome sequence of Dysgonia stuposa is available in the Supplemental Files and at GenBank: MK262707.
