# Supplementary material for: The complete mitochondrial genome of Dysgonia stuposa (Lepidoptera: Erebidae) and phylogenetic relationships within Noctuoidea
Source: PeerJ. 2020 Mar 16;8:e8780. doi: 10.7717/peerj.8780 (PMC7081777; doi:10.7717/peerj.8780)
Supplement: Supplemental Information 2 [file peerj-08-8780-s002.docx]

> *Dysgonia stuposa*

TTAAAAATAAGCTAAGATAAGCTTTTGGGTTCATACCCCAACTATAAAGGATAAACCTTTTTTTAAAAAAAATAAAGTGCCTGATTAAAGGATTATTCTGATAGGATAAATTAAGTAAATTATTATTTACCTTTATTATTTTTTTTTTATATTTTATAGAATTAAACTATACCTATTAGTATCAAAAACTAATGTGCATCATACACCAAAATATAATTTTTTATAATAATGAATTTTCAATTCTTTTAAATTTTAATTTTTTTATTTTAAATTCTTTTTTCTTTTAACTCCTCAAAAATATTTTTTTTTTTTATTTTAATTTTTAGAACATTAATTTCAATTTCAGCAAATTCTTGAATTGGATGTTGAATTGGTTTAGAAATTAATTTATTAAGATTTATCCCCCTAATTTCTAATTCTAATAATTTATTATCTACAGAAGCATCTTTGAAATATTTTCTAACACAATCTATTGCATCTATTAATTTTTTATTTTCAATTTTAATAAAAATAATATTATTAAAAAATTTTGAATTAAATTTTTTTTTATCTATTATAATTAATTCATCAATATTAATAAAAATAGGAGCTTCCCCCTTTCATTTTTGATTCCCCAATATTGTAGAAGGTTTATCATGATTTAATAATTTTATTTTAATAACTTGACAAAAAATTACCCCCATAATTATTTTGTCTTATTATTTTAATAAAAATTTTATTTTAATAATTATTATATTAAATGCTATTATTGGAGCTTTAGGAGGATTAAATCAAACTTCATTACGTAAAATTATAGCATTTTCTTCCATTAATAACTTAAGTTGAATATTATCATCTATTTTAATTAGAGAAAATCTATGATTATTTTACTTAATTATATATTCATTTATAATTAGAATTTTATGCTCCACTTTTTATTTTTTAAATGTTTTTTTTATTAATCAATTATTTATTAACAACATAAATTCCCTAATTAAAATTAATTTATTAATTAATTTTTTATCATTAGGAGGTTTACCCCCTTTTATTGGATTTTTCCCCAAATGAATTATTATTAACTTTTTAATTATAAATCAAATATATTTTTTAACTTTTATTTTAATTATAATAAGATTAATTTTACTTTTTTTTTATATTCGTATTATCTATTCAACTTTTATATTCAATTATTTTAAAATAAAATGATTTAAAATTTATATTAAAAATAATAAATTTACACTAATTAATATTTTTTCCTTTTTCTCTCTTTCAGGAATAATTCTTAGAACCTTTTTTTTCTTATAAGGTTTTAAGTTAAATTAAACTAATAGTCTTCAAAATTATTTATAAAGAGATATTCTTTAAGCCTTAGTATTGTTTAATTTACTCCTTAAAATTTGCAATTTTATATCATTATTGAATATAAGGCTTTTTAATTATCTTAAAACTTATTAATAAAAGAGAAAACTCTCGTAAATAAATTTACAATTTATCGCTTATTCCTCAGCCATTTTATTTTTTTTTTGCGAAAATGACTTTACTCAACAAATCATAAAGATATTGGAACTTTATATTTTATTTTTGGTATTTGAGCAGGAATAGTAGGAACTTCTCTAAGTTTATTAATTCGAGCAGAATTAGGAAATCCAGGATCACTAATTGGCGACGATCAAATTTATAATACTATCGTTACGGCTCATGCTTTTATTATAATTTTTTTTATAGTTATACCAATTATAATTGGAGGATTTGGTAATTGATTAGTACCTTTAATATTAGGAGCTCCAGATATAGCATTCCCTCGAATAAATAATATAAGTTTCTGACTTCTTCCCCCTTCTCTAACTCTTCTTATTTCAAGAAGAATTGTAGAAAACGGAGCAGGAACTGGATGAACCGTATATCCTCCACTATCTTCTAATATTGCACATAGAGGAAGATCAGTAGATTTAGCTATTTTTTCCCTACATTTAGCTGGAATTTCATCCATTTTAGGAGCTATTAATTTTATTACTACAATTATCAATATACGATTAAATAGTTTAATATTTGATCAAATACCACTATTTGTTTGAGCAGTAGGAATTACAGCTTTTCTACTTTTATTATCTCTACCTGTTTTAGCTGGAGCTATTACTATACTTTTAACAGATCGAAATTTAAATACTTCATTTTTTGACCCAGCTGGAGGAGGAGATCCTATTCTTTATCAACATTTATTCTGATTTTTTGGACACCCTGAAGTTTATATTCTAATTCTTCCAGGATTTGGTATAATTTCTCATATTATTTCACAAGAAAGAGGAAAAAAAGAAACATTTGGATGTTTAGGGATAATTTATGCTATACTAGCTATTGGATTATTAGGATTTATTGTATGAGCACATCATATATTTACTGTAGGAATAGATATTGATACTCGAGCTTATTTCACATCAGCAACAATAATTATTGCTGTACCTACAGGAATTAAAATTTTCAGATGATTAGCAACCTTTCATGGTACACAAATTAACTATTCTCCTTCAATTTTATGAAGTTTAGGATTTGTATTTTTATTTACAGTAGGAGGATTAACTGGAGTAATTTTATCTAATTCATCTATTGATATTACTTTACATGACACTTACTATGTTGTAGCTCATTTTCACTACGTATTATCTATAGGAGCTGTATTCGCAATTATAGGAGGATTTATTCATTGATATCCTTTATTTACAGGATTATGTTTAAATCCTTATTTATTAAAAATTCAATTTTTTATTATATTTATTGGAGTTAATTTAACCTTTTTTCCTCAACATTTCCTAGGATTAGCTGGAATACCTCGACGATATTCTGATTATCCAGACTCTTATATTTCTTGAAATATTATTTCTTCTCTAGGATCTTATATTTCATTATTAGCTGTTATACTTATATTAATTATTATTTGAGAATCAATAATCAATCAACGAATTGCTCTATTCTCATTAAATTTACCTTCTTCAATTGAATGATATCAAGCTCTTCCACCTGCTGAACATTCATATAATGAACTTCCTATTTTAAGAAATTTCTAATATGGCAGATTATATGTAATGGATTTAAACCCCATTTATAAAGGTTTATCCTTTTTTTAGAAATAGCAACATGATCTAATTTTAATTTACAAAATAGAGCTTCCCCTTTAATAGAACAAATTATTTTCTTTCATGATCATACTTTAATTATTTTAATTATAATTACAATTCTAGTTGGTTATTTAATAGTAAGATTATTATTTAATAAATATATTAATCGATTTTTATTAGAAGGTCAAATAATTGAATTAATTTGAACAATTTTACCAGCAATTACTTTAATTTTCATTGCTCTTCCCTCTTTACGTTTACTCTATTTATTAGATGAATTAAATAATCCTTTAATTACTTTAAAATCTATTGGCCATCAATGATATTGAAGTTATGAATATTCTGATTTTCATAATATTGAGTTCGATTCTTATATAATTCCATCAAATGAATTACAACCTAATAACTTTCGATTACTAGATGTTGATAATCGTATTATTTTACCAATAAATAATCAAATTCGTATTTTAGTAACAGCAACTGATGTTATTCACTCATGAACTGTCCCATCTTTAGGTGTCAAAGTAGATGCTAACCCAGGTCGATTAAACCAAACTAATTTTTTCATTAATCGACCTGGAATTTTTTATGGTCAATGCTCAGAAATTTGTGGAGCAAATCATAGTTTTATACCTATTGTAATTGAAAGAATCTCAATTAAAAATTTTATTAATTGAATTAATAATTATTCTTCATTAGATGACTGAAAGCAAGTACTGGTCTCTTAAACCATTTTATAGTAAATTAGCAATTACTTCTAATGAAAAGAATTAGTTAAATTTATAACATAAATATGTCAAATTTAAATTATTACATTAGTAATATTCTTTTATCCCTCAAATAATACCTATTAATTGATTAATATCTTTTTTCTTTTTTATTTGTATTTTTTTAATTTTTAATATTATAAATTATTATATTTATAATATTAATATTAATAATACAGATAATAAATTAAATATTAAAAAAAAAAATATCAATTGAAAATGATAAGTAACTTATTTTCAATTTTTGACCCTTCTACTAATATTTTTAATATTTCTTTAAATTGAATTAGAACAATTTTAGGAATTTTATTTATTCCTTATTCATTTTGATTAATCCCTAATCGTCATTTTATGTTTTGAAATTTTATTTTATCTAAACTTCATAATGAATTTAAAACTTTATTAAAAAATAATTATTTCCAAGGATCAACATTTATTTTTATTTCAATATTTACATTCGTCTTATTTAATAATTTTTTAGGTTTATTCCCTTATATTTTTACTAGAACAAGTCATTTAACCCTTTCATTATCAATCTCTTTACCTTTATGATTGAGATTTATAATTTATGGATGATTAAACAACTCCCAACATATATTTATTCATATAATTCCTCAAGGAACACCCTCAGTTTTAATACCTTTTATAGTCTTAATTGAAACAATTAGTAATATTATTCGACCAGGAACATTAGCAGTTCGATTAACAGCTAATATAATTGCTGGACATCTATTAATAACATTACTTAGTGGAACAGGACCTAATATAAATCATTATATAATTATATTATTAGTATTAATTCAAATTTTATTATTAATTTTAGAATCAGCAGTTGCGGTTATTCAATCCTATGTTATTGCAATTTTAAGAACTTTATATTCTAGTGAAGTAAATTAACTTTAATTAATTAATATTTATAAATTAATGAAAATTACCCATAATCACCCATTTCACTTAGTTGATTACAGACCATGACCCCTAACAGGAGCTATTGGTGTTATAACTTTAGTAACTGGAATAGTTAAATGATTCCACAACTTTAATTTAAATTTATTAATTTTAGGATATATTATTGTTATTTTAACAATATATCAATGATGACGAGACGTTTGTCGAGAAGGAACTCTTCAAGGTAAACACACTATTTTAGTAACTAAAGGACTTCGATGAGGTATAATTTTATTTATTGTATCAGAAATTTTTTTTTTTATTTCTTTTTTTTGAGCATTTTTCCACAGAAGTTTATCTCCTAATATTGAAATTGGATCTATATGACCACCTACAAGTATTACTCCTTTTAACCCATTTCAAATTCCCCTTCTTAATACTATTATTTTAATTAGATCAGGAGTATCAGTTACCTGAGCTCATCACGCCTTAATAGAAAATAATAACTCTCAAACAACTCAAGGTTTATTTATCACTATTATTTTAGGAATTTATTTTACAATTCTTCAAGCTTACGAATATTTCGAAGCTCCTTTTACTATTGCAGATAGAATTTATGGATCTACTTTTTTTATAGCAACAGGATTCCATGGATTACATGTTATTATTGGAACTTTATTTTTATTAATTTGCTTAATTCGCCATTTAAATAACCATTTTTCTAGAAATCATCATTTCGGATTTGAAGCAGCTGCATGATATTGACATTTTGTAGATGTAGTTTGATTATTCCTTTATATTTCTATTTACTGATGAGGAAATTAATTATTTATATAATATATTTAGTATATTTGACTTCCAATCAAAAAGTTTAAAAATTTTTAATATAAATAATTATTCTTATAATAAGTATTTTCACATTAATTACATTAATCTCTAATATTATAATATTTCTTTCCATTATTTTATCAAAAAAATCATTTTCAGATCGAGAAAAATCATCCCCATTTGAATGCGGATTTGACCCTAAATCTTCTGCTCGTATTCCATTTTCCCTTCATTTTTTTTTAATTACAGTTATTTTTTTAATTTTTGATGTAGAAATTGCACTAATTTTCCCAATTATTCCATTATTTAAAATAGTAAATTTTTTTCTATGAACAAAAATTAGTTTTTTTTTCTTAATTATTTTAATTTTTGGTCTTTATCATGAATGAAATCAAAATATATTAAACTGAACAAATTAAATAATATATATATATATATATATATATATATATGGAGAATAGTTTATAAAAACATTTGATTTGCGCACACAAAAAAAAGATAGTTTTCTCTCTCTCTTTTTTATATATAAAAAAAAAAATATATTTTTTTTTATATATATATATATATATATATATATATATATATATATATATATATATATATATATAAATAAATAAATAAGAAGCAAATTTTGCATTTAATTTCGACTTAAAAGATTGAGTTAAATAAACTCCTTATTTATTATATTTACTTAATTGAAACCAAAACAGAGGTATATCACTGTTAATGATAAAATTGAATAATAATTCCAATTAAATTTAATTAGAAATATAAAGTTTAAAAATAAGCTGCTAACTTAATTTTTAGTGGTTAAATTCCATTAATATTTCTTCATATATATATATATATATATATATATATATATTTATATAGTTTAAACCTAAAACTTTACATTTTCATTGTAAAAATAAAAATTTCTTTTTTATAAATATATATATATATATATATATATATATATATATATATATATATATATATATATATTTAAAAATAATAACTATTTCCTTAATATCTTCAATATTATGCTCTATTTTATAAGCTATTTAAATATAATAATAATAATATAAATAAGCTAATTATTATTCAAATAATAAATCTAAATAAATAAATTTTTAAACTATTTATCTGAAAAAAATTATAAAAAATAGAATACTTTTTCATAATTATTATTAAACCTCATCCACTATAAACTTCTCTTCAACCTATATCAATATTTTTTAATAAATTTTGACCAAAATTAAGAAAATAATATCTTAACCCATAAGTTGATAAATTAGGTATAAATCATATTATACATAAAAAATTTCTTATATTATATCTTATTAAAAATTTATTAACAGAATAAATTTCTATATTTCTTACTAAATAACCTATAATTCCACCAATAATTCTAACATAAATTACTATTATTTTTATATTAAAAGGTAAATAAATTATATAAGGATAAGAAAAAATTATTCATCTTAAAAATCTACCTCTAATAACTCTTATAAATAATAATGTAAATATACTTTTTAATATAATATAATCCTCATCATATAAATTATAAACTCTAATTAAATTAAAATCTCTTACTATTAAATATATAATTAATCGAATTGTATAAAATATTGTTAATCCTGTAGAAACATAGTATAATAAAAAAATTAATAAATTTAAATTTCTAAATCTAACTAATTCTAAAATTAAATCCTTAGAATAAAATCCAGCTAAAAATGGAATTCCACATAAAGCTAAATTAGAAATATTTATACATAAAGATGTTAAAGGAATATAAAATCTAATTCCCCCTATAAAACGAATATCTTGTATATCATTTATTATATGAATAATTACACCAGCACATATAAATAATAAAGCTTTAAATATTGCATGAGTTAATAAATGAAAAAAAGCTAAATCAGGTATTCCCATACTTAAAATTCTTATTATTAACCCTAATTGTCTTAATGTAGATAAAGCAATAATTTTCTTTAAGTCAAACTCATAATTAGCTCTAATACCAGCTATGAATATAGTTAATCCCGATAATAATAATAAAATTTTTAAAAAAAACGTATCTAATAATAAAAAATTAAAACGAATTAATAAATAAACCCCAGCAGTAACTAAAGTAGATGAATGGACTAATGCTGAAACTGGTGTAGGAGCAGCTATAGCCGCAGGTAATCATGAACTAAAAGGAATTTGAGCTCTTTTTGTTATAGCAGCTATAATAATTATTCTTCTAATTATAATCATTTCTCAATCATTTTTTATAAATTCTAAATAAAAAATATAGTTTCATCTACCATAATTTATTATTCAAGAAATAATTAATAAAATAAATACATCCCCAATACGATTTGATAATGCAGTTAACATTCCAGCATTATAAGATTTTAAATTTTGATAATAAATAACCAACAAATAAGAAACTAATCCTAATCCATCCCAACCTAATAAAATTCTAATAATATTAGGACTAATAATTAATAATATTATAGAAGTTACAAATAATAAAACTAAAATAATAAAACGACTTAAATTTAATTCTGATCTTATATATCTTTTTCTATAATAAATAACAACAGAAGAAATTAAAAAAACAAATATTATAAATAATAATGATATTCAATCTAATAAAATAGACATTACAATACTCATTGAATTAAAAGAAATAATTTCTCATTCTAAAAAAATAACTATATTATTTATAATAAAATAAATCATCATAAAAAAATTTAATAATCTTAATATTATTAAAAAAAAAAAAGAAATAAAACAAATAGAATATTTTAAATTATTTATAATTTAAAATGAAATTTTATTCACATTTTTGACACCACAAATCAATATTTTTATTAAATTATTTAAATAAAATCAAATTATTCTATAATCAATTTTAATTACTATTAAATTTAATGGTAATCAATGTAATATTAATATTAAATATTCACGTGAAACACCAGTATAATATCTATAAATACCTGAATAATATTTTCCATGTTGTACATAAGAATATAAATATAATCTATAACCGGCACTAAAAAAAGAAATTAATATTAATATAATTATAGATAATCAAGATCATCTCACTAATCTATTAATTAAACTAATTTCACCTATTAAATTTAAACTTGGAGGAGCAGCTATATTAGAAGATATTAATAAAAATCACCATAAACTTATAGAAGGTATGAAATTCATTATTCCTTTATTAATATATAATCTTCGACTATGTAACCGCTCATATCTAATATTAGCTAAACAAAATATTCCAGAAGAACATAATCCATGACCGATTATTATAATATAAGAACCTAAAAACCCTCAATAATTTATAATTATAATACCTCTAATCACTAATCTTATATGAGCAACAGAAGAATAAGCAATTAATGATTTAATATCAACTTGACAAAAACATTTTAATCTAATATAAAATCCACCTACTAATCTAATAACAATTCTAATATAATTTAACTTTAAATTTACCTGCTGTAAAAAAATTATCAAACGTAATAATCCATACCCCCCTAATTTTAATATAATCCCAGCTAAAATTATAGAACCTGAAACTGGAGCTTCTACATGAGCCTTAGGTAATCATAAATGAACAAAATATATAGGTATTTTAACTAAAAAAGCTATAATTATACAAAAATATAATATATATATATCTATATTAATAAATTTTAAAAAATAAATTATTATTCTATTTATCTCATTAAAAATATAAAAAATTCCTATTAATAAAGGTAAAGAAACAAATAAAGTATAAAATAATAAATATATTCCAGCCTTAATTCGCTCTGGTTGATAACCCCACCCAATAATTAATATTAATGTAGGAATTAATCTACCCTCAAAAAATAAATAAAATATAAATATATTTATAACACTAAAAGTTAAAAATAATATAATTAATAAAAAAATAATATTAAAAATAAAAAAATTAACATAAAAATTTACCTTATAAATATTTTCTCTAGCTATAATTATTAAAACAGAAATTCAAATTCTTAACAAAATTAAACCATATGACATAATATCACAAGATAATATATAACTTAAATTACAAAATAAACCAAATCTTATTATTATATTTATATATATAAATATCATCAAAAATAATATTATTTGAACCATTCAAAATATATTTCTCATAAAACATATTGGTAAAATAAAAATTATTATTATTAAAAATTTTATCATTTTTTTTTTTATTTAATCAATAAAATCCAATTTTATTATAAATTATAAAAGATTAAATCCTTGAAAATAATCATTACCGTGAGTACGAATTAGAGATACTAAAATTGATAAACCTAAAGCACCTTCACAAACAGAAAAAACTAAAAATACTATTAATATATATATATCATATTCAATATATCTTAAAAAAATTAATAAAAAAAAAAAAATTCTTAATACAATAAATTCTAATCTTAATAAAACAATTAATAAATGCTTATGTTTTAACACAAAAATTATATTACCAGTAATAAATATAATAATAAAAATTAATCATATAAATATTATCATTTATAATTTAAATTGTTTTTATAGTTTAAAAAAAACATTGGTCTTGTAAATCAAAATTAAGTATTTTACTTTAAAAACTTCAAAGAAAAAGAATATCTTTATCAATAATCTCCAAAATTATTATTTTAATTAAACTATTCTTTGATTTGATATAACAAAAATATTTTTATCTTTTATAATCATATTAATTTCATTTTTTATAATATTTTTAAATAATCCCCTATCAATAGGATTAATAATTTTAATTCAAACATTATTAATATGTTTATTATCAGGTATATTAATTAAAACTTATTGATTCTCTTATATTCTTTTTTTAACTTTCCTAGGAGGATTATTAGTATTATTTATTTATGTATCAAGAATTGCTTCAAATGAACTTTTTAAACCCTCATTTAATGCAAAATTAGTATTTATTTTTTCACTAATAATCTTATCTATCATCCAAATTATTTTCATAAATAATCTATTTTGAATAAATTTTTCATTCAACTCAGATATAGACAATTTTTACATATTATCATTATTTATTAATAATGAAAATAAAATTAACTTAAGTAAATTATATAATAATCAAACCTTTATAATTATATTAATATTAATTATCTATTTATTTATTACATTAGTAACAGTAGTAAAAATTACAAATATTTTTTATGGACCTTTACGATCTAAAATATAAAAATTTAAAATATAATGACAAACAATAATAACAATAAATTCATCCTAATACGAAAAACTAATCCTATTTTTAAAATTCTAAATGGATCATTAATTGATTTACCCTCCCCTTCAAATATTTCTTATTTATGAAATTTTGGTTCATTATTAGCATTATGTTTAATTATTCAGATTCTAACAGGACTATTTTTAACAATATATTATACAGCTAATATTGAATTAGCATTTTATAGAGTTAATTATATTTGCCGAAATGTAAATTATGGATGATTAATCCGAACCCTTCACGCTAATGGAGCATCTTTTTTTTTTATTTGCATTTATATTCATATTGGACGAGGAATTTATTACGAATCCTTTAACCTAAAACATACATGAATAATTGGAGTAACAATCTTATTTTTATTAATAGCAACTGCTTTCATAGGATATGTTTTACCTTGGGGTCAAATATCATTTTGAGGAGCAACTGTTATTACTAATTTATTATCAGCAATTCCTTATCTAGGATCTATATTAGTAAATTGAATTTGAGGAGGATTTTCAGTTGATAATGCAACTTTAACCCGATTTTATACTTTTCATTTTTTATTACCATTTATTATTTTAATAATAACTATAATTCATTTACTTTTTCTACACCAAACAGGATCTAATAATCCATTAGGATTAAATAGAAATTACGATAAGATTCCATTTCATCCATTTTTTTCTTATAAGGATTTATTAGGAGCCATTATATTATTATTTATATTAATTATATTAACTTTAACTAATCCTTATTTATTAGGAGATCCAGATAATTTTATTCCAGCTAACCCATTAGTAACACCTGAACATATTCAACCAGAATGATATTTCTTATTTGCCTACGCTATTTTACGATCAATTCCTAATAAATTAGGAGGGGTAATTGCCTTAGTTATATCAATTTTAATTTTAGTTATTTTACCCTTTACTTTTAATAAAAAAATTCAAGGAATTCAATTTTATCCTTTAAATCAATTTCTTTTTTGAACTTTAGTAACAATAATTATTTTATTAACATGAATTGGAGCTCGACCAGTTGAAGATCCTTATATTATTACAGGACAACTACTTACTATTTTTTATTTTTCATATTTTATCATCAACCCTTTAGTAAATATATACTGAGATAATTTAATTTTTAATTAATTAATGAGCTTGTATATTAAGCATTTGTTTTGAAAACTTAAGAAAGAATAAAAATTCTATTAATTTATACTAAAAATAATCAACAAAATTATATTAAAAAAATTTTTAAACCTAAAAAAAATAATAAAAAATTTATAGAAACAGGCAAATAACTCTTTCAAGCTAAATATATTAATTTATCATAACGATAACGAGGTAAAGTCCCCCGCACTCAAATAAATAAAAAAGAAATAAAAGTTAATTTTAAATAAAAAAAAATTCTTAAATTATAACCCCCTATATAAATTATAACAAATAATAATCTTATAAATAAAATACTAGAATATTCAGATAAAAAAATCAATGTAAACCCACCTCTTCTATATTCAACATTAAATCCTGATACTAACTCTCTTTCTCCTTCAGCAAAATCAAATGGAGTTCGATTAGTTTCTGCTAATATTGATCTAATTCAACATAACCCTAAAGGAAACATTAAAATAATAAATCAAACTATATTTTGATAATAAAAAAAACTTAATAAATTATAATCTATCACTATAACAATACTTGACAGTAAAATTATTGCTAATCTTACTTCATAAGAAATAGTTTGAGCAACAGCACGTAACCCTCCTAATAAAGCATAATTTGAATTAGATGATCAACCAGCAACCATAACTGTATAAACCCCTATTCTTATACAACATAAAATAAATATAACCCCTAAATTAAATCTAATTAAATTAAAATAATAAGGAATAACTATTCAAATTAATAAAGATAAAGTAAAACCAACAACAGGAGAAAAATAATACATAATATAATTAGAATAATTTGGATAAGTTTGCTCCTTAGTAAATAATTTAATAGCATCTGCAAAAGGTTGTAAAAGACCTATAATCCCCAATTTATTTGGACCTTTACGAATTTGAATATAACCTAATACTTTACGTTCTAATAAAACTAAAAAAGCAACTCCAATTAAAACCCCAATAATTAAAATTAATAAACCAATAAAAATTATATAAATATCATATATTATCATATACTATCTATATAAATTAATTATACATTTATGACTTCTAAAATCATTACATTTTTCTGCCAAAATAGCTTCTAAATATATATATATATATATATATACTATTTATAACTAATTTAATTAATAATTTTTAACCTAATTAATAAAATTCGAATAAAAAAAAATTTAATTTAAATATTTAATCCTTTCGTACTAAAATATTTCCTTTTAAAAGATAGATACCAACCTGGGCTCACACCGGTTTGAACTCAGATCATGTAAGATTTTAATGATCGAACAGATCAAAATTTTAAACTTCTGCATTTAAATTTTATCTTAATCCAACATCGAGGTCGCAAACTCTTTTTTTTATTTGAACTAAAAAAAAAAATTACGCTGTTATCCCTAAGGTAATTTTTTCTTATAATCAATAAAATTGGATCACTTTTTCACTTATTTATGTTCATATTTTAAAAAAAGTTATTTTTATTTTTCTATCACCCCAACAAAATAATTAAATAAATTAAAATTTAATTTTATTTATAAATAATTTTAATTTATTTAACTATAAAACTCTATAGGGTCTTCTCGTCTTTTTAATTCATTTTAACTTTTTAATTAAAAAATTAAATTCTATAATTTAATTAAGAGACAGATTATATTTCATCCAATCTTTCATACAAGTCACCAATTAAATGACTAATGATTATGCTACCTTTGTACAGTCAATATACTGCAGCCCTTTAATTATAAAATCAGTGGGCAGATTAGACTTTATATTATTTACAAAAAGACATGTTTTTGATAAACAAGTGAATATATATATTTGCCGAATTCCTTTTAATTAATTTAATTTTAATAAATAAATCTATTTAAAACTAACTATATACTAATTTTATCATTATAACTAATTTTAAAATATTTAAAATTAATTTTTTATAAAAAAAATAAATTTTTAATTAAATTTTATTTTTAATGAAATTATTTATAATAAATTAAAATTTATTAACAATATAAATTATATAATTTTCAAATAATTTAAAATAATACCTATTATATTTATTTAATTTAAAGATTATCCCTTAAATTATAAAATTTAAATATTTATTAATTAATTAATTAATTAATAAAATAAAATAAATTTAAAATTAAATTTTTTTCTAAAAAAACTAGATATATTTAAGAACGATTAACATTTCATTTCCAATTAATTATTAAAAATATTTATGCCACAATAACTTTTTTAATTAATTATCTCTCTTAAATTCGAGAATTATTAAATCCAAAAATATTTTTTAATAAACTCTGATACACAAGATACACTAAATAAAAATTACTTTTAAATAAATTTTTATTTAAATTATTTCAAAATTCTTTCACAATACTAATTCACTATAAACTTTTAAATTATTTCTATTAAAATACTTTAACCCCCATTAAAATAATTTTAATTTTTTAAATTTAAAAATTTTTTTATTATTACTTATTTATTAATTTTTCCCCTCAAATTAATTAATTTTTATAATATCTTTTCAATGTAAATGAAATACTTTATCTCAAGCTCTAATTTGTTCTTTCTAGAAACACTTTCCAGTACCTCTACTTTGTTACGACTTATTTCAACTTTAAATTATATGAAAGCGACGGGCAATATGTACATATTCTAATTTAAAATCATTTTATTAAATTAAATAAAATTACATTTAAATCCAATTTCAATTAATTTTTCCAAATTAATATTCATTTAAATAAATTTATTGTAATCCATTATATTCTTAATTATAATCTGCATCTTGATCTGATTTAATTTTATTAATAATTTTTAAATATTACCTTTATTAAAAAATATTTTTTTAACAACGATATACAAAATTATAAATTAAGTAAATTTATTCGTGGATTATCAATTATTAAACAGATTCCTCTAAATGAACTAAAATACCGCCAAATTGTTTAAGTTTCAATAAATAATTAATTACTATTTTAGTATTTTTAATTTAAAATTTTAATAATAGGGTATCTAATCCTAGTTTTTAAAAAAATTTATTAAATCATAAAATCTTAAATAATATTTTATTAAATTAAAAATTTCACCTAATAATTTAAAATTTATATTATATTTTATTATTATTAATTAATTTACTAATAAAATTTAATTTAATCTTTGTTTAACCGCAACTGCTGGCACAAAATTAGTTATTAATTTAAATATTACTAAATATTAATTTCTTAAATATTTTAATATTAATTACTAAAAAAAAATTAATTTATTATTTAAATAAAGAAATATTAACACTAAAATTTATATGTAAAATAAACTTTAAATAAATTTTCCAAACTACAAAAATTTTTATTTATATGCACAATTTCTCACATAGATTTTTTTTTTTTTTTTTTTTATATTTAAATATTTATTATATAATATTATTTTATATTAAAATATTTAATATAATTATTAAATATTAAATAATTTCTTTTTCTTTTTTCTTCATACTATTCATATTGAAACCTAATTTGGAAATTAAACAATTACAATTCTTAAAAATTACAATATATTAATATAATTAATAATAATTTTTCTTAATAAGTTAATGAATTATAAATATTTTAATTTATTTAAAAATTTAATATATATATATAAATATTAATTTTATAAAAATTTAATATATATATATATATATATAATTTTAAAGAAAATTATTATTTAATTATGTATTTAAACCATTTTTAATAATAATGCATATAAATAAAAAAAAATA
